# Synergistic fusion of a multilevel visual transformer in CNN for variable-length volumetric radiographic data analysis and content-based retrieval

**DOI:** 10.1038/s41598-026-53594-1

**Published:** 2026-05-22

**Authors:** Muhammad Owais, Muhammad Zubair, Taimur Hassan, Divya Velayudhan, Naoufel Werghi, Irfan Hussain

**Affiliations:** 1 Khalifa University Center for Autonomous Robotic Systems (KU-CARS), Khalifa University, Abu Dhabi, United Arab Emirates; 2 School of Computer Science, University of Galway, H91 TK33 Galway, Ireland; 3 Department of Applied Artificial Intelligence and Robotics, School of Computer Science and Digital Technologies, Aston University, Birmingham, United Kingdom

**Keywords:** Synergistic deep learning, Medical image analysis, Computer-aided diagnosis, Volumetric radiographic data, Medical content retrieval, Pattern recognition, Infectious diseases, Biomarkers, Medical research, Engineering

## Abstract

Volumetric radiographic data analysis poses significant challenges due to its 3D structure and variable input lengths. Moreover, the unpredictable distribution of diseased regions, often spanning multiple slices and interspersed with normal tissue within abnormal volumes, further complicates the analysis. Despite advancements, existing 3D volumetric analysis methods predominantly rely on 2D slice selection and expert intervention, limiting scalability and efficiency. Additionally, a prevailing challenge is harmonizing the analysis of volumetric radiographic data with variable length. To address these limitations, we introduce a novel deep learning framework that synergistically fuses a lightweight multilevel vision transformer with a convolutional neural network (CNN). The proposed approach independently extracts and aggregates spatial features from 2D slices while preserving multilevel contextual information. A second-stage recurrent module is further integrated to handle variable-length inputs by leveraging single annotations for complete 3D volumes and exploiting their structural features. Empirical validation of our method is conducted on a composite of three publicly accessible radiographic repositories, demonstrating superiority (p-value $<$ 0.01) over existing alternatives. The results achieved highlight remarkable metrics: 98.54% accuracy, 98.51% F1-score, 98.77% average precision, and 98.25% average recall. To facilitate further research and development, we will publicly release the proposed framework and associated resources, providing a robust foundation for future studies. The implementation and materials is available at our GitHub.

## Introduction

In the realm of medical image analysis, volumetric data plays an essential role, offering substantial applications in pathology diagnosis, computer-aided diagnosis (CAD), surgical planning, and research. Volumetric imaging techniques such as Computed Tomography (CT), Magnetic Resonance Imaging (MRI), and Optical Coherence Tomography (OCT) provide a three-dimensional (3D) perspective of anatomical structures. Through efficient data analysis, these techniques enable the identification of regions of interest, thereby enhancing the accuracy and depth of medical interpretations compared to traditional two-dimensional (2D) imaging methods^1,2^.

The CT scans, particularly volumetric radiographic lung CT scans, are crucial in the diagnosis and management of pulmonary diseases^3^. These scans provide high-resolution, cross-sectional images of the lungs, enabling the detection and characterization of various lung conditions, including tumors, nodules, interstitial lung disease, and infections such as pneumonia and tuberculosis. The 3D nature of these scans allows for detailed visualization of lung anatomy and pathology, facilitating accurate assessment and monitoring of disease progression. Recent advancements in volumetric lung CT imaging have significantly improved the sensitivity and specificity of lung cancer detection^3,4^. For instance, the application of low-dose CT screening in high-risk populations has been shown to reduce lung cancer mortality by enabling early detection of small nodules that may not be visible on traditional chest X-rays^5^. Efficient analysis of volumetric lung CT data through state-of-the-art AI techniques enhances the identification of regions of interest, thus improving the accuracy and depth of medical interpretations. For example, AI-driven CAD systems can automatically detect and quantify the size and growth of pulmonary nodules, assisting in the early diagnosis of lung cancer and other pulmonary diseases^4^. These systems have demonstrated remarkable success in clinical settings, offering valuable second opinions and reducing the workload of radiologists^6^.

The paradigm of recent artificial intelligence has demonstrated significant and promising potential in redefining the landscape of proficient CAD tools, particularly within the realm of volumetric radiographic data^7,8^. These state-of-the-art solutions primarily leverage deep learning algorithms for the analysis of radiographic data sourced from different modalities, such as CT and X-ray scans, contributing to enhanced diagnostic accuracy and treatment outcomes^9^. Deep learning algorithms can extract intricate features from data, offering valuable insights into specific diseases. Despite their success, the dynamic field of deep learning undergoes continuous evolution to refine performance and improve precision and diagnostic efficacy for medical data^10^. In a broad spectrum of medical applications, input data is intricately presented as 3D volumetric images (CT and MRI), offering detailed anatomical information^11^. These volumetric images effectively encapsulate the essence of two-dimensional image sequences, each characterized by a variable number of frames or slices. Furthermore, these sequences are annotated by assigning the same label to each respective slice or sequence of an image.

### Potential research gaps and motivation

Despite substantial advancements, a persistent challenge exists in effectively aligning 3D medical imaging data analysis, characterized by varying lengths^12^. This challenge arises from the inherent diversity within such data, where the number of frames or slices may fluctuate from one instance to another. Achieving consistency and deriving meaningful insights across this spectrum of data lengths necessitates innovative techniques that seamlessly adapt to these fluctuations. Another significant challenge with existing CAD methods for volumetric data lies in accurately selecting relevant slices from the entire 3D volume. This process is complicated by the unpredictable distribution of diseased regions, which often span multiple slices and are interspersed with normal tissue, necessitating considerable manual effort and time. These are some limitations with existing image-based CAD methods: 1) Limited spatial context: These methods examine only one image at a time, increasing the likelihood of overlooking crucial spatial context. This limitation makes it challenging to localize targeted regions or lesions that extend across multiple images^13^. 2) Poor Generalization: The outcome of image-based methods is influenced by the selection of slice orientation and thickness in radiographic data^14^. 3) Annotation bias: Conventional CAD systems are biased by their reliance on annotated training slices, limiting the exposure of the model^15^. In contrast, volumetric data considers all slices, offering a more comprehensive view of the anatomy. They possess advantages in better generalization and precision towards lesion regions compared to 2D CAD approaches. Moreover, they are less sensitive to slice thickness and orientation variations and are not as influenced by the choice of annotated slices^16^. Addressing this challenge has the potential to refine the diagnostic and prognostic capabilities of medical imaging systems and extend their applicability across a broader array of scenarios and patients with varying data characteristics.

### Main contributions

This study presents a novel synergistic deep learning framework to overcome the challenges encountered by slice-based approaches in diagnosing lung infections using CT volumetric data. A multilevel spatio-recurrent attention network (MSR-AN) is designed to tackle the challenge of variable-length 3D medical imaging data. The proposed model comprises multilevel spatial-attention sub-network (MSA-SN) and recurrent feature alignment sub-network (RFA-SN) to extract multilevel spatial and 3D structural features, enhancing diagnostic performance. The framework facilitates the analysis of individual slices and accommodates sequences of slices with varying lengths. This adaptive functionality substantially enhances the model capacity, enabling it to handle various clinical scenarios and contribute to more comprehensive diagnostic assessments. Quantitative evaluation illustrates a significant enhancement in performance compared to state-of-the-art techniques.

The notable contributions of this study can be summarized as follows:

1. The proposed MSA-SN synergistically integrates a lightweight multilevel visual transformer encoder and a convolutional neural network (CNN), where the transformer encoder captures long-range dependencies and the CNN extracts local spatial details, enabling diverse feature extraction and robust representation learning.
2. We introduced a volumetric feature alignment stage (RFA-SN) that imparts a hybrid characteristic to our model. This feature equips our model to make diagnostic decisions using individual slices or sequences of varying lengths.
3. To enhance interpretability, we extend the framework to a volumetric medical content retrieval system, allowing retrieval of similar cases from a database to support clinical decision-making.
4. The framework is publicly accessible to support reproducibility and future research via GitHub repository (MSR-AN).

The subsequent sections are arranged as follows: Section II reviews related work, while Section III explains the methodology of the proposed framework, delving into aspects such as model structure, design, and workflow. Section IV comprehensively outlines the experimental configuration, results, and analysis, encompassing both quantitative and qualitative dimensions. Finally, Sections V and VI encapsulate the discussion and conclusion of this study, respectively.

## Related work

This section comprehensively analyzes the best-performing techniques for classifying medical diagnostic data. Patients’ diagnostic imaging samples are relatively small, posing a challenge for deep learning techniques. Deep learning models, being high-capacity models, typically require a substantial amount of data for effective training. Transfer learning techniques are employed to overcome this limitation. Therefore, we review transfer learning-based techniques that fine-tune pre-trained models on medical imaging data.

### 2D image-based deep learning techniques

In the realm of 2D diagnostic imaging data, previous studies have explored various CAD methods employing pre-trained CNN models. Kaur et al.^17^ utilized a pre-trained VGG16 model^18^ to classify pathological and healthy brain images. However, the method validation relied on a limited dataset of 20 normal and 140 abnormal MRI images. Similarly, Ashraf et al.^19^ employed GoogleNet^20^ to classify medical images across 12 categories, with a total dataset of 3600 images but a restricted sample size of 300 samples for each class. Akpinar et al.^21^ utilized a parameter-efficient deep learning model, SqueezeNet, in a diagnostic system designed for chest disease classification. The diagnostic system was validated using 660 X-ray images. Aloyayri et al.^23^ conducted breast cancer classification and achieved superior results by coupling the potential of ResNet18 through transfer learning on histology images. Souid et al.^24^ focused on chest lesions, introducing a multiclass diagnostic framework using the lightweight MobileNetV2 CNN model^25^. Their extensive single-modality dataset comprised 64,699 images to validate the model performance.

Similarly, skin lesion classification was undertaken by Jasil et al.^26^ and Çakmak et al.^27^. Jasil^26^ employed DenseNet201^28^ for dermoscopic image classification with a limited dataset of 3,091 images, while Çakmak^27^ used NASNetMobile^29^ for melanoma detection with a larger dataset of 10,015 images. In another study, a CAD system was developed by Gambhir et al.^30^ for the classification of severity levels of diabetic retinopathy (DR). In this regard, the potential of ShuffleNet^22^ was exploited to differentiate DR into different severity levels. Collectively, these studies contribute to exploring CAD methodologies for diverse medical imaging applications, showcasing the effectiveness of pre-trained CNNs across different modalities and pathologies.

Recent advancements in medical imaging research have yielded notable breakthroughs in detecting COVID-19 and emphysema. Ahuja et al.^7^, presented a pioneering study focused on COVID-19 lung infections employing a sophisticated three-phase model, integrating transfer learning from CT scan images, data augmentation, and pre-trained CNN models like ResNet18. The ResNet18 model demonstrated exceptional performance, showcasing superior classification accuracy during training, validation, and testing. Another study by Elpeltagy et al.^8^ addressed the urgent need for precise COVID-19 detection by leveraging transfer learning from ResNet50 and introducing modifications for enhanced discrimination. This proposed model achieved remarkable accuracy rates for CT scans and X-ray images, outperforming alternative approaches and highlighting its potential as an invaluable automated tool for COVID-19 diagnosis. Wu et al.^9^ also tackled emphysema classification challenges using a Vision Transformer (ViT) model, achieving impressive accuracy and surpassing other prominent models. These studies underscore the evolving landscape of medical imaging, demonstrating the effectiveness of advanced models in addressing critical challenges in disease detection and classification.

### 3D sequence-based deep learning techniques

Limited research exists on developing a CAD system tailored for the analysis of 3D diagnostic images. In sequence-based data analysis, predominant research has embraced the integration of Long Short-Term Memory (LSTM) in conjunction with CNN^31^. Srinivasu et al.^31^ pioneered a framework for skin lesion classification, wherein a sequence of images, rather than a single image, is input to MobileNetV2^25^ and LSTM for disease classification. Similarly, Ebrahimi et al.^32^ focused on Alzheimer disease detection. They developed a CAD system for 3D brain MRI scans, leveraging the cascade of Residual Net^33^ and LSTM models, and employed a substantial dataset comprising 35,550 MRI samples for system development and evaluation^34^.

In the domain of volumetric CT data, Kollias et al.^10^ introduced AI-based methods for COVID-19 detection and severity analysis. Their approach integrates a deep learning model based on ResNet50 and the LSTM network, showcasing its effectiveness in COVID-19 disease detection. Arsenos et al.^12^ contributed a CT database for COVID-19 (COV19-CT-DB) diagnosis and an innovative deep-learning model for disease diagnosis. Their model, RACNet, integrates 3D analysis through a CNN-RNN network. RACNet accommodates CT scans of varying lengths through dynamic routing, feature alignment, and a mask layer. Furthermore, Owais et al.^13^ proposed a deep 3D volumetric model for efficient lung infection screening via chest CT scans, utilizing ShuffleNet and LSTM. In the CT imaging-based disease analysis domain, Gupta et al.^14^ introduced a pulmonary nodule classification framework that uses a CNN and LSTM model to classify volumetric CT data into benign or malignant classes. In a separate study, Zhang et al.^15^ developed a model for categorizing pathological subtypes of pulmonary nodules on CT images. This study predicts pathological subtypes by evaluating the correlation of imaging features with the pathological characteristics of pulmonary nodules.

### Constraints of existing techniques

The idea of attention-based multilevel feature aggregation has seen minimal exploration within 3D diagnostic imaging data analysis for classification tasks. Various information fusion methodologies, depending on the aggregation stage and learning strategy, have been proposed, such as early fusion stage and ensemble learning^35^. These approaches have proven effective in enhancing the performance of deep learning (DL) models. However, it is important to note that these methods often necessitate additional preprocessing and post-processing operations, contributing to the overall increase in computational overhead and parameter expansion. Moreover, recent multiscale attention-based medical imaging frameworks such as RDNet^41^ and hierarchical refinement with adaptive deformation cascaded models^42^ further highlight the effectiveness of hierarchical feature refinement and multi-scale attention strategies for improving structural representation in medical image registration. Similarly, recent attention-enhanced and transformer-based medical image segmentation frameworks^46–48^ further exhibit the effectiveness of multiscale feature refinement and advanced representation learning strategies in improving structural understanding across medical imaging tasks.

In a study by Abdar et al.^35^, a conventional ensemble learning approach was employed to leverage the advantages of multi-level feature fusion. The proposed feature extraction scheme involves utilizing four distinct pre-trained models, collectively encompassing 162 million trainable parameters, thereby demanding substantial computational resources. Acknowledging that such computational demands may limit the feasibility of implementation in resource-constrained environments. Moreover, numerous investigations have employed image-based models, focusing solely on the spatial information of 3D imaging data during diagnostic decision-making. This approach may introduce a limitation, as the exclusion of vital 3D anatomical information could lead to erroneous predictions and ultimately reduce the overall predictive confidence of testing data outcomes. Therefore, future research efforts should explore methodologies that effectively integrate spatial and anatomical information for a more comprehensive medical 3D imaging data analysis.

### Singularity of the proposed method

**Fig. 1:**
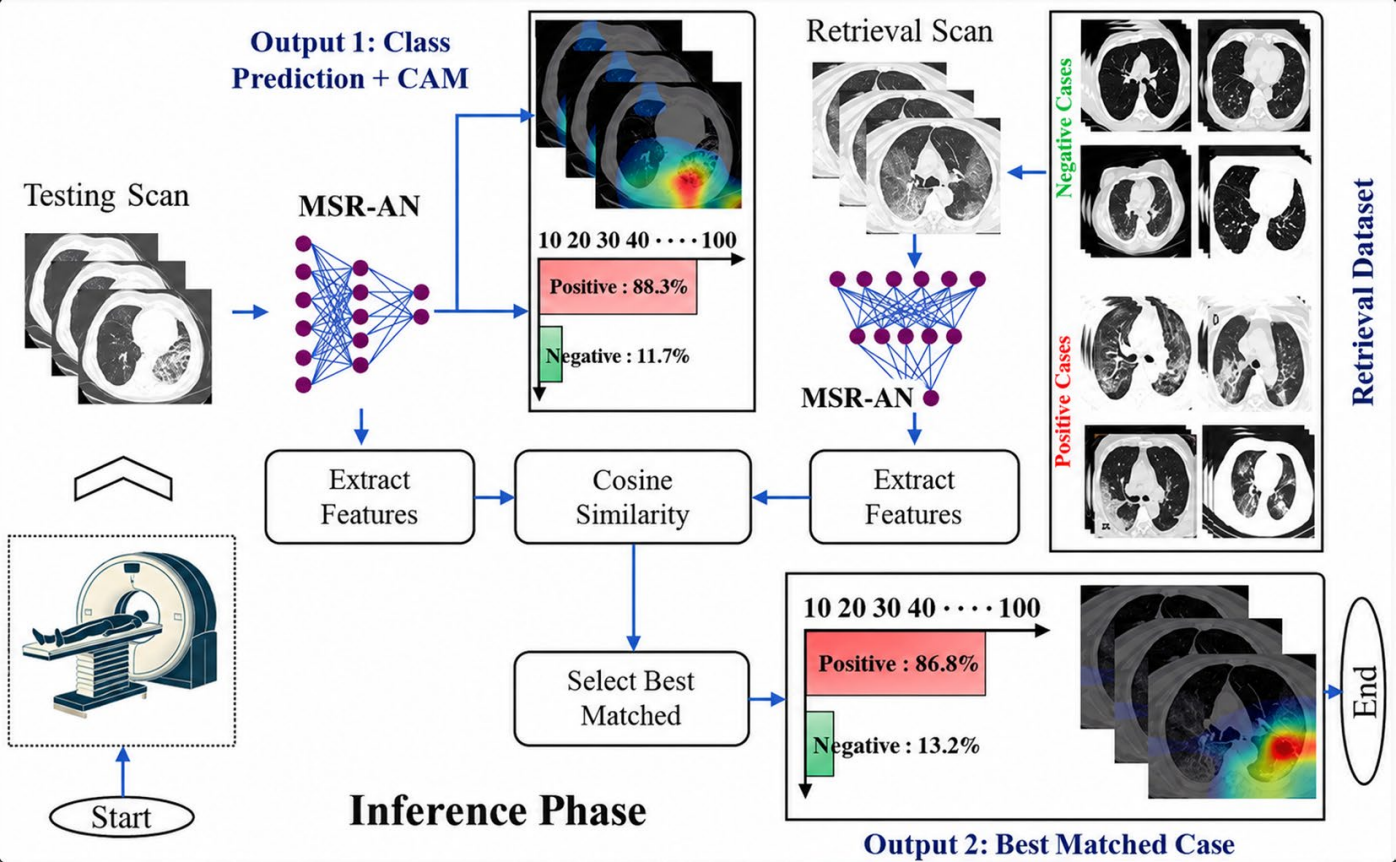
A volumetric-based medical content retrieval framework demonstrates the extended application of the proposed model to enhance the interpretability of CAD decisions.

In the context of reshaping computer-aided diagnostic tools in the medical domain, our proposed method stands out as a significant breakthrough. It adeptly tackles the challenge of analyzing variable-length 3D medical imaging data, eliminating the need for manual image selection or expert guidance. Our presented model couples the benefits of transfer learning in categorizing 3D imaging data, thereby eliminating the requirement for excessive training parameters. Notably, the model yields promising results for 2D and 3D imaging data, showcasing its proficiency in classifying sequences within 3D imaging data. Empirical validation, conducted on publicly accessible repositories, further underscores the substantial potential of our approach in advancing automated lung infection detection. This firmly establishes our proposed method as a valuable contribution to medical diagnostics. Furthermore, we extend the proposed model by developing a volumetric-based medical content retrieval framework to enhance its interpretability and clinical relevance. As shown in Fig. 1, the framework systematically retrieves the most relevant past cases from a medical database based on volumetric similarity and diagnostic relevance. Leveraging advanced feature extraction and deep learning-based similarity measures, it identifies diagnostically significant cases, aiding healthcare professionals in cross-referencing and validating decisions. The process begins with the proposed classification model, which accurately categorizes cases, followed by a retrieval pipeline that selects and presents similar past cases, enhancing diagnostic reliability and decision validation.

## Proposed method

**Fig. 2:**
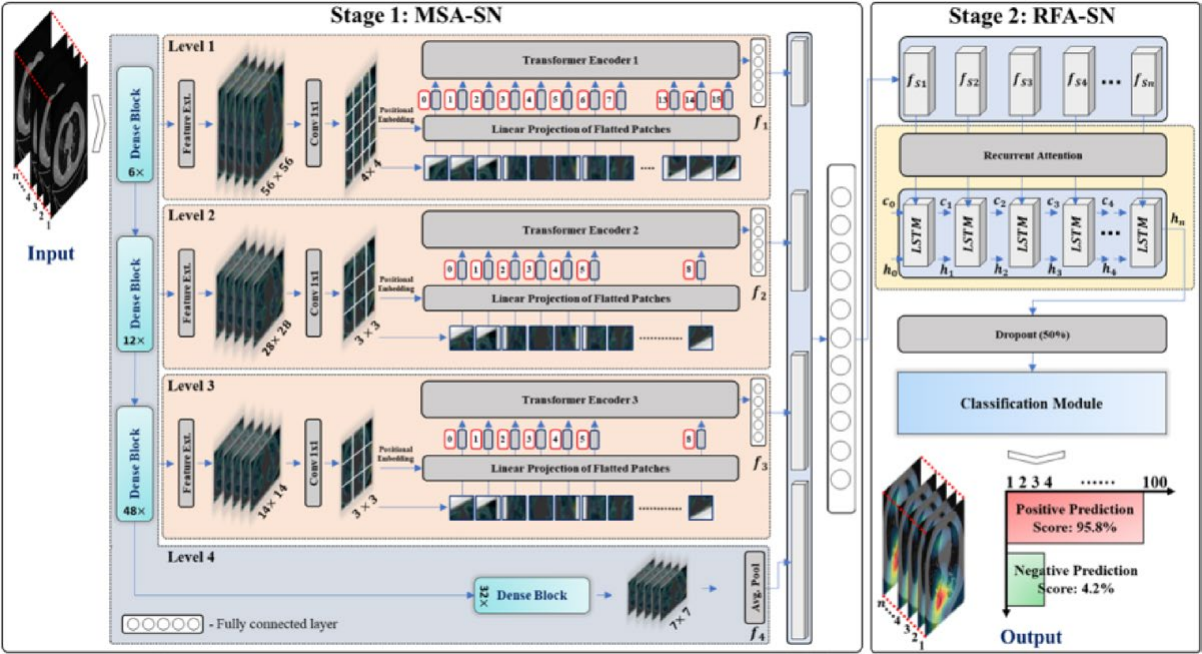
Architectural overview of the proposed multilevel spatio-recurrent attention network (MSR-AN): Integrating spatio-attention multilevel feature aggregation and recurrent volumetric feature alignment for enhanced performance.

The architectural design and configuration of the proposed MSR-AN model are illustrated in Fig. 2. The proposed network comprises a dual-stage architecture. The initial stage, the MSA-SN, focuses on extracting multi-scale spatial features from each 2D input slice. Subsequently, the second stage, referred to as the RFA-SN, adeptly addresses variable input lengths through singular annotations encompassing the entire 3D inputs. This stage further harnesses three-dimensional structural features of the input sequence of n successive slices ${{(i.e.,\;\boldsymbol{S}}}_{1},{{\boldsymbol{S}}}_{3},{{\boldsymbol{S}}}_{3},\dots ,{{\boldsymbol{S}}}_{{\boldsymbol{n}}})$, resulting in precise decision-making. A detailed explanation and workflow for each stage are presented in the following subsections.

### Multilevel spatio-attention sub-network

To attain superior classification performance, the initial MSA-SN model mainly focuses on extracting multi-scale spatial features from each 2D input slice by utilizing the strength of a lightweight multilevel ViT and CNN to exploit diverse features in the spatial domain. It also utilizes the attention mechanism to concentrate on the spatial aspects of the input data. Subsequently, it aggregates features from various levels of abstraction to enhance its overall performance. The attributes of the spatio-attention block, combined with dense blocks^33^, are attached to aggregate multilevel spatial context within each slice effectively. The key intuition behind fusing CNN-based dense blocks with transformer-based spatio-attention blocks is to leverage their complementary strengths. CNNs capture local spatial features and patterns, while transformers effectively model long-range dependencies and global context. Combining them allows the model to benefit from detailed local feature extraction and broad contextual understanding, resulting in a more robust and comprehensive feature representation.

1. **Spatio-Attention Block:** The configuration of the proposed spatio-attention block (SA-block) is based on the attention mechanism (illustrated in Fig. 2), widely used in deep learning models to highlight specific regions of input data that are more relevant to the task. The attention mechanism captures object-specific patterns and spatial relationships by assigning importance to particular regions within the input image/slice. The SA block is designed to capture spatially endorsed low-level details and high-level semantic information by aggregating features from multiple levels of abstraction. Mathematically, it processes the input tensor ${{\boldsymbol{X}}}_{i}\in {\mathcal{R}}^{{w}_{i}\times {h}_{i}\times {d}_{i}}$ by harnessing additional attention-driven features and giving a new output tensor $\boldsymbol{X}_{j} \in {\mathcal{R}}^{{w_{j}  \times h_{j} \times d_{j} }}$:
  1 $${\mathcal{H}}_{{SA - Block}} \left( {X_{i} ,\varphi } \right) = g_{{\varphi _{k} }} \left( {\mathop A\limits^{ \circ } _{i} \left( {h_{{\varphi _{i} }} \left( {X_{i} } \right)} \right)} \right)$$
2. In the given context, the notation ${\mathcal{H}}_{SA-Block}\left(\cdot \right)$ represents the spatio-attention block, functioning as a transfer function. The functions ${h}_{{\boldsymbol{\varphi }}_{i}}\left(\cdot \right)$ and ${g}_{{\boldsymbol{\varphi }}_{i}}\left(\cdot \right)$ correspond to convolutional and fully connected layers, respectively, with trainable parameters ${\boldsymbol{\varphi }}_{i}$,. The notation ${{\AA }}_{i}\left(\cdot \right)$ is used to denote the self-attention layers that assign weights to different parts of the input tensor, enabling the model to focus on relevant image regions while making predictions. Generally, the attention score is calculated by transforming the input sequence of vectors i.e., ${\boldsymbol{X}}=\left[{{\boldsymbol{x}}}_{1},{{\boldsymbol{x}}}_{2},\dots ,{{\boldsymbol{x}}}_{n}\right]$ into three new vectors: query (${\boldsymbol{Q}}$), key (${\boldsymbol{K}}$), and value (${\boldsymbol{V}}$)^16^. Then, the attention score is calculated as follows:
  2 $$Attention\left({\boldsymbol{Q}},{\boldsymbol{K}},{\boldsymbol{V}}\right)=\mathrm{s}\mathrm{o}\mathrm{f}\mathrm{t}\mathrm{m}\mathrm{a}\mathrm{x}\left(\frac{Q{K}^{T}}{\sqrt{{d}_{k}}}\right)V$$
3. Where ${d}_{k}$ is the dimension of the key vector that stabilizes the gradients during the training process.
4. **Dense Blocks:** The proposed MSA-SN architecture integrates dense blocks derived from a dense network backbone[28]. These blocks are specifically crafted to tackle the vanishing gradient problem during training, facilitating the convergence of the network to optimal solutions. The efficacy of these dense blocks is evaluated through experimental ablation, which involves training MSA-SN without them. Consequently, we couple the advantages offered by dense blocks in our proposed MSA-SN architecture. The mathematical expression for a dense block can be defined as follows: ${{\boldsymbol{X}}}_{i}={h}_{{\boldsymbol{\varphi }}_{i}}\left(\left[{{\boldsymbol{X}}}_{0}\oplus{{\boldsymbol{X}}}_{1}\oplus,\dots ,{\oplus{\boldsymbol{X}}}_{i-1}\right]\right)$, here, ${{\boldsymbol{X}}}_{i}$ represents the output of the i-th layer within the dense block. The concatenated outputs of all previous layers within the same dense block are denoted by $\left[{{\boldsymbol{X}}}_{0}\oplus{{\boldsymbol{X}}}_{1}\oplus,\dots ,{\oplus{\boldsymbol{X}}}_{i-1}\right]$. The function ${h}_{{\boldsymbol{\varphi }}_{i}}\left(\cdot \right)$ is a composite function involving sequential convolution operations, batch normalization, and activation functions. Within each dense block, another hyperparameter exists, which is known as the growth rate. To represent this relationship for the i-th set of convolutional layers within each dense block, the expression is formulated as follows: ${k}_{i}={k}_{0}+k\times \left(i-1\right)$. Here, the growth rate of the network is specified by $k$, the input depth of the feature map ${{\boldsymbol{X}}}_{0}$ is defined by ${k}_{0}$ and ${k}_{i}$ represents the resulting depth of the i-th set of convolutional layers.
5. Finally, transition layers are employed to reduce the spatial dimensions of the feature maps ${{\boldsymbol{X}}}_{i}$ and control the number of feature maps transferred from one dense block to the next, effectively managing computational complexity.
6. The mathematical equation for a dense block incorporating a transition layer can be defined as follows: ${\mathcal{H}}_{DE-Block}\left({{\boldsymbol{X}}}_{0},\boldsymbol{\varphi }\right)={\mathrm{T}}_{i}\left({{\boldsymbol{X}}}_{i}\right)$, here, ${{\boldsymbol{X}}}_{i}$ represents the output of the last layer within the preceding dense block. ${T}_{\mathrm{i}}\left(\cdot \right)$ denotes a composite function of the transition layer, which regulates the depth and dimension of ${{\boldsymbol{X}}}_{i}$ by applying 1 × 1 convolution operation and 2 × 2 average pooling layer. This helps in managing computational complexity and improves the flow of information through the network. The notation ${\mathcal{H}}_{DE-Block}\left(\cdot \right)$ designates the dense block utilized as a transfer function.
7. **Multilevel Feature Aggregation:** In the proposed MSA-SN model, we applied a multilevel feature aggregation scheme that extracts semantic features at low-, intermediate-, and high-level (notated as ${{\boldsymbol{f}}}_{1}$–${{\boldsymbol{f}}}_{4}$) that can capture fine-grained details as well as more abstract, high-level patterns. These multiscale features are aggregated to collectively contribute to the model outcome using multiple spatio-attention blocks, as illustrated in Fig. 2. This comprehensive representation is crucial for tasks requiring understanding local and global contexts. These multilevel features are selected based on four distinct spatial sizes (i.e., 56 × 56, 28 × 28, 14 × 14, and 7 × 7) of dense block output tensors. This selection ensures a comprehensive capture of details, with each size representing different levels of feature abstraction. This range ensures that features from small local details to larger global patterns are adequately captured, which is crucial for tasks requiring multiscale feature representation. This fusion process (i.e., ${\boldsymbol{f}}=Fusion\left({{\boldsymbol{f}}}_{1},{{\boldsymbol{f}}}_{2},{{\boldsymbol{f}}}_{3},{{\boldsymbol{f}}}_{4}\right)$) ensures a rich and varied representation for each class, enhancing the model ability to learn subtle patterns and characteristics within images. A detailed ablation study (covered in a subsequent section) provides concrete evidence of how the incorporation of multilevel feature aggregation significantly contributes to achieving state-of-the-art performance. This study underscores the effectiveness and importance of this approach in advancing classification accuracy and overall model capabilities.
8. **Model Training Scheme:** First, the input slice undergoes transformation through a convolutional layer employing 64 filters sized 7 × 7 × 3. As the channel dimension expands, a max-pooling layer is utilized to manage the spatial dimension, creating a down-sampled feature map with dimensions of 56 × 56 × 64. Subsequently, a stack of four dense blocks, incorporating three transition layers (as depicted in Fig. 2), sequentially operates on the preceding output and produces a feature map of size 7 × 7 × 1920. Ultimately, a high-level feature vector ${\boldsymbol{f}}_{4}$ is obtained, having dimensions of 1 × 1 × 1920, by utilizing a 7 × 7 average pooling layer on the output feature map. Additionally, a stack of SA-blocks was employed to extract multiscale and multi-level semantic features. These dense blocks are chosen for their ability to provide diverse information and output tensors with varying spatial dimensions (i.e., 56 × 56, 28 × 28, and 14 × 14), enabling the capture of both low- and intermediate-level semantics. An FC layer is employed to globally consider all multilevel semantic features (i.e., ${\boldsymbol{f}}_{1}$–${\boldsymbol{f}}_{4}$) and generate discriminative patterns. As a result, an output feature vector ${\boldsymbol{f}}$ with dimension 1 × 1 × 256 is generated, representing multilevel semantic information. This process yields a set of n feature vectors $\left\{ {{\boldsymbol{f}}_{{{\mathrm{S}}1}} ,{\boldsymbol{f}}_{{{\mathrm{S}}2}} ,{\boldsymbol{f}}_{{{\mathrm{S}}3}} \ldots ,{\boldsymbol{f}}_{Sn} } \right\}$ with dimensions 1 × 1 × 256 × n for consecutive slices (${\boldsymbol{S}}_{1} ,{\boldsymbol{S}}_{2} ,{\boldsymbol{S}}_{3} \ldots ,{\boldsymbol{S}}_{n}$). Finally, RFA-SN is applied to the resultant feature vectors for the generation of additional volumetric features.

### Recurrent feature alignment sub-network

1. **Model Structure:** In our second-stage RFA-SN model, we adopt a multi-step approach that combines the power of self-attention mechanisms with recurrent neural networks (RNNs) to effectively handle volumetric data. An essential advantage of incorporating a self-attention mechanism in volumetric data analysis is its capability to utilize inter-sequence attention maps. These attention maps enable the model to proficiently consider complex relationships and dependencies among the feature vectors of n successive slices (${{\boldsymbol{S}}}_{1},{{\boldsymbol{S}}}_{2},{{\boldsymbol{S}}}_{3}\dots ,{{\boldsymbol{S}}}_{n}$), ultimately leading to enhanced performance and a deeper understanding of the data, particularly in the medical domain. Subsequently, an LSTM model^34^^31,32^ is employed as the RNN model to systematically analyze the inter-slice attention maps, considering the context of both preceding and succeeding slices. LSTM is selected as the RNN model for our second-stage RFA-SN due to its following potential advantages: 1) addressing the vanishing gradient issue, ensuring stable training and the ability to learn intricate patterns in volumetric data, 2) providing transfer learning capability without significantly impacting overall training parameters. This strengthens the model performance in classifying volumetric data, especially in medical applications, and 3) handling the sequential nature of the data, adapting to different sequence lengths. Since the LSTM processes sequential feature embeddings extracted from individual CT slices, it inherently supports arbitrary-length volumetric inputs by aggregating inter-slice contextual dependencies through its memory states and producing a unified volume-level prediction.
2. **Workflow:** The working scheme of the proposed RFA-SN is represented in Fig. 2. Initially, a set of $n$ feature vectors (i.e., $\left\{{{\boldsymbol{f}}}_{\mathrm{S}1},{{\boldsymbol{f}}}_{\mathrm{S}2},{{\boldsymbol{f}}}_{\mathrm{S}3}\dots ,{{\boldsymbol{f}}}_{Sn}\right\}$) are transformed through a sequence input layer and assigned to the self-attention layer. This layer generates inter-sequence attention maps by highlighting the importance of each vector based on its contextual information within the sequence of feature vectors. Following this, the LSTM layer capitalizes on 3D structural dependencies within these inter-sequence attention maps, ultimately yielding a singular feature vector ${{\boldsymbol{h}}}_{n}$ sized 1 × 1 × 1200. This output feature vector ${{\boldsymbol{h}}}_{n}$ encapsulates both 2D and 3D spatial as well as structural information corresponding to the entire sequence of slices (${{\boldsymbol{S}}}_{1},{{\boldsymbol{S}}}_{2},{{\boldsymbol{S}}}_{3}\dots ,{{\boldsymbol{S}}}_{n}$). The extracted information undergoes further refinement through a fully connected (FC) layer to leverage more discriminative patterns. Finally, the classification module (based on FC, SoftMax, and classification layers, as shown in Fig. 2) makes the sequence-based diagnostic decision for the given sequence of n slices (${{\boldsymbol{S}}}_{1},{{\boldsymbol{S}}}_{2},{{\boldsymbol{S}}}_{3}\dots ,{{\boldsymbol{S}}}_{n}$).

### Training loss

A two-step training strategy was employed for both the MSA-SN and RFA-SN models, performed in a sequential manner to achieve better convergence of the entire network. In the first step, the focus was on effectively joining spatial features $\langle {[{{\boldsymbol{S}}}_{T}]}_{i=1}^{p},{[{label}_{T}]}_{i=1}^{p}\rangle$ from the complete training dataset by training an MSA-SN model through transfer learning using weights of the pre-trained DenseNet were applied to dense blocks ${\mathcal{H}}_{DE-Block}\left(\bullet \right)$ (∙) within the MSA-SN. In the subsequent step, both the training and validation datasets underwent transformation denoted by $\langle {[{{\boldsymbol{f}}}_{Tr}]}_{i=1}^{p},{[{GT}_{Tr}]}_{i=1}^{p}\rangle$) and $\langle {[{{\boldsymbol{f}}}_{Va}]}_{i=1}^{q},{[{GT}_{Va}]}_{i=1}^{q}\rangle$), respectively. This transformation function applies the MSA-SN model to each data sample. Following this, the second RFA-SN model was trained to acquire an understanding of the 3D structural dependencies inherent in the complete volume data. The mathematical formulation of our two-step loss function can be expressed as:

3 $${\mathcal{L}oss}_{total}=\left\{\begin{array}{c}\underset{{w}_{DN}^{^{\prime}}}{arg min} {\mathcal{L}}_{1}\left({\psi}_{DN}\left({w}_{DN},{\left[{{\boldsymbol{S}}}_{Tr}\right]}_{i=1}^{p}\right),{\left[{GT}_{Tr}\right]}_{i=1}^{p}\right) \\ \underset{{w}_{MSA-SN}^{^{\prime}}}{arg min} {\mathcal{L}}_{1}\left({\psi}_{MSA-SN}\left({w}_{MSA-SN},{\left[{{\boldsymbol{S}}}_{Tr}\right]}_{i=1}^{p}\right),{\left[{GT}_{Tr}\right]}_{i=1}^{p}\right) \\ \underset{{w}_{RFA-SN}^{^{\prime}}}{arg min} {\mathcal{L}}_{2}\left({\psi}_{RFA-SN}\left({w}_{RFA-SN},{\left[{{\boldsymbol{f}}}_{Tr}\right]}_{i=1}^{p}\right),{\left[{GT}_{Tr}\right]}_{i=1}^{p}\right)\end{array}\right.$$

where ${\psi}_{MSA-SN}$ and ${\psi}_{RFA-SN}$ as transformation functions represent the MSA-SN and RFA-SN models, respectively. ${\mathcal{L}}_{1}(\bullet )$ and ${\mathcal{L}}_{2}(\bullet )$ are the CE loss functions. The sequential two-step training approach allows previously trained blocks to be frozen in the complete model, which helps stabilize the learning process and prevents the disruption of learned features.

### Code availability

The code developed and used in this study to generate the reported results is publicly available to ensure transparency and reproducibility. The full implementation, including model architecture, training procedures, and evaluation scripts, can be accessed at: GitHub

## Results and analysis

### Dataset

**Fig. 3:**
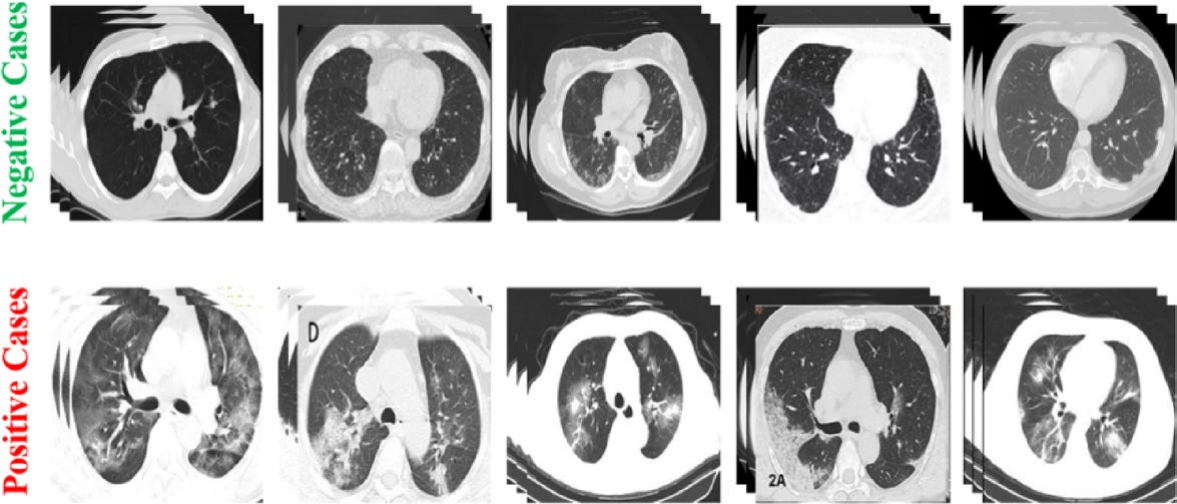
A visual overview of a few selected 3D scans for each class from the used dataset. The first and second rows depict samples from negative and positive classes, with columns showcasing distinct cases within each class.

Chest CT scans were used to evaluate the proposed framework by merging three publicly available datasets: BIMCV COVID-19^43^, COVID-CT^44^, and The Cancer Imaging Archive (TCIA)^45^. The combined dataset contains 5,471 CT scans from 2,789 distinct patients, comprising 3,254 infected scans (1,660 patients) and 2,217 normal scans (1,129 patients). Representative examples of infected and normal CT slices are shown in Fig. 3. To confirm reliable evaluation and prevent data leakage, dataset partitioning was accomplished using patient-level separation, meaning that scans from the same patient were not distributed across training, validation, and testing subsets. The dataset was divided into 70% training, 10% validation, and 20% testing samples while preserving class balance across splits, as summarized in Table 1. Since the dataset was created by combining multiple public repositories, duplicate samples were assessed using metadata consistency checks available from each dataset source. Furthermore, the proposed framework was evaluated using fivefold cross-validation, ensuring balanced representation of infected and normal samples across folds while maintaining strict patient-level separation. This dataset formation strategy ensures fair evaluation and prevents overlap between training and testing samples, improving the reliability of the reported performance results.

**Table 1:** Summary of the lung CT datasets used in this study, including number of images and patients in the infected and normal classes, and the patient-level data splitting protocol for training, validation, and testing.

Dataset	Infected (Images / Patients)	Normal (Images / Patients)
BIMCV COVID-19^43^	2905 / 1311	-
COVID-CT^44^	349 / 349	397 / 397
TCIA Cancer Archive^45^	-	1820 / 732
**Total (Combined)**	**3254 / 1660**	**2217 / 1129**
Training (70%)	2278 / 1162	1552 / 790
Validation (10%)	325 / 166	222 / 113
Testing (20%)	651 / 332	443 / 226

### Experimental protocol

The simulations for both the proposed and baseline models were conducted using the MATLAB (R2023b) coding framework, integrating the deep learning toolbox. The computations were performed on an Intel Core i7 CPU Desktop computer equipped with 64 GB RAM and an NVIDIA GeForce GTX 4090 graphics processing unit (GPU). To control overfitting, an independent validation dataset, denoted as $\langle {[{{\boldsymbol{S}}}_{Va}]}_{i=1}^{q},{[{GT}_{Va}]}_{i=1}^{q}\rangle$ was employed. Additionally, parameter selection and the training-stopping criterion were defined based on the validation dataset. The training process concluded upon achieving the highest validation accuracy, as detailed in Algorithm 1. A five-fold cross-validation strategy was implemented, allocating a ratio of 70:10:20 for the training, validation, and test sets, respectively, to ensure generalization. Prior to training, all CT slices were preprocessed using contrast-based intensity normalization using *imadjust(.)* function in MATLAB, followed by resizing to the required network input resolution (e.g., 224 × 224 for CNN and 384 × 384 for ViT models). No lung-region cropping or segmentation was applied in order to preserve full anatomical context. Since the data were collected from multiple public repositories with heterogeneous acquisition settings, variations in slice thickness, in-plane resolution, and scanner protocols were handled through slice-wise feature learning within the proposed framework. The same preprocessing pipeline was applied consistently across all folds to ensure reproducibility. Further dataset-specific acquisition and reconstruction details can be found in the original sources: BIMCV COVID-19^43^, COVID-CT^44^, and The Cancer Imaging Archive (TCIA)^45^.

### Evaluation protocol

The performance was analyzed using a set of six top performance evaluation metrics commonly employed in medical image analysis. The assessment encompasses True Positive Rate (TPR), True Negative Rate (TNR), Accuracy (ACC), F1 Score (F1), Average Precision (AP), and Average Recall (AR). The performance of the proposed model is accessed by contrasting it with state-of-the-art techniques and baseline models through these metrics. In addition, statistical significance was assessed using paired two-tailed t-tests across five cross-validation folds, where fold-level performance metrics were used as analysis units. Two significance levels were considered: 99% confidence (p < 0.01) and 95% confidence (p < 0.05), confirming consistent comparison between the proposed and baseline models.

**Algorithm 1:**
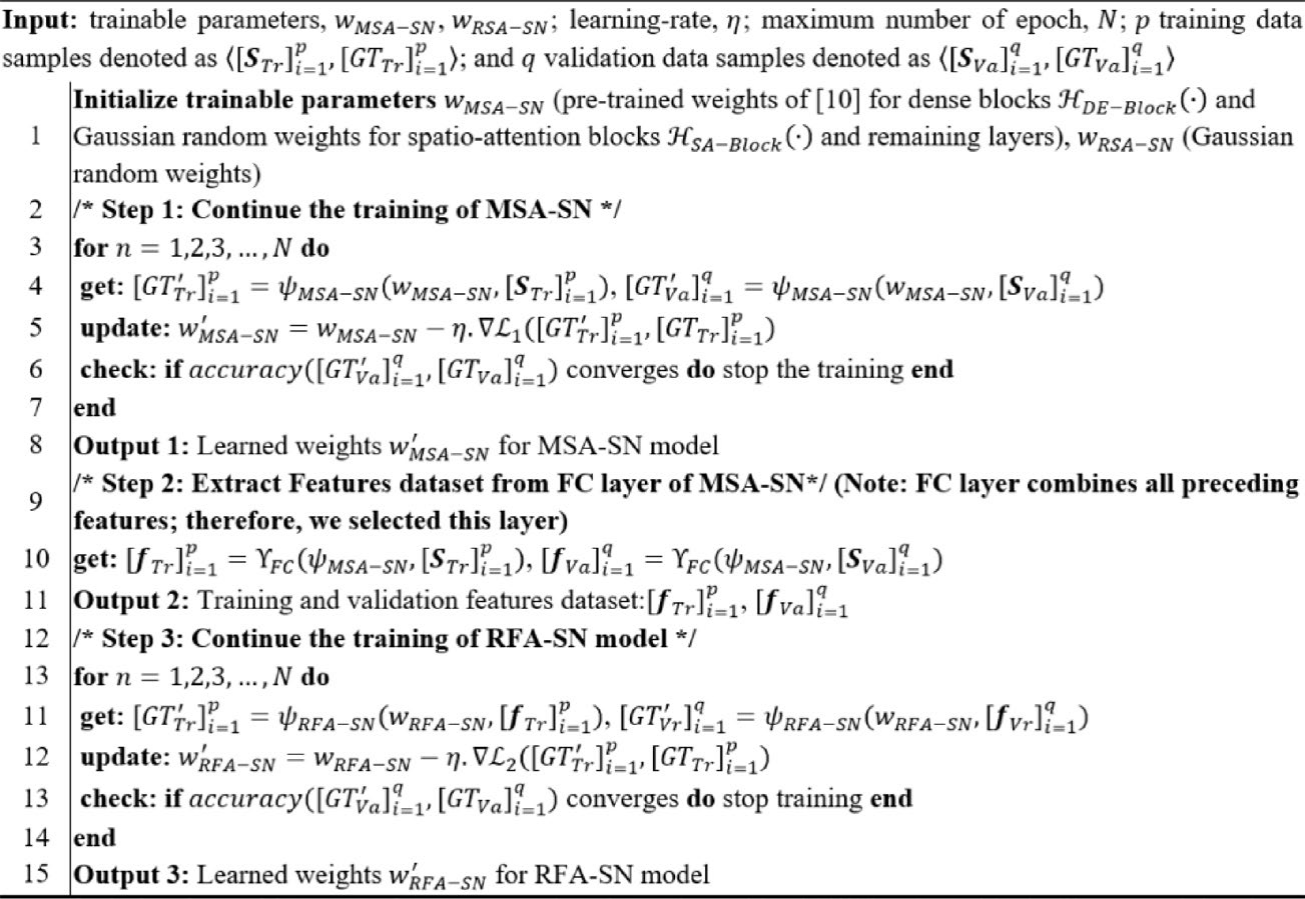
Two-step sequential training.

### Quantitative assessment and comparison with baseline

The quantitative outcomes of the best-performing proposed MSR-AN model, along with both the second-best performing model ${\psi}_{MSA-SN}$ and the baseline mode ${\psi}_{\mathrm{D}\mathrm{N}}$, are analyzed in Table 2. The experiments were systematically conducted to ensure the effectiveness of the proposed architectural design. The comparative assessment protocol is outlined as follows: 1) In the initial evaluation of ${\psi}_{MSA-SN}$ and ${\psi}_{\mathrm{D}\mathrm{N}}$, we treated the dataset as 2D images, neglecting the inherent 3D anatomical dependencies in volumetric scans. 2) In the subsequent comparison (${\psi}_{\mathrm{M}\mathrm{S}\mathrm{A}-\mathrm{S}\mathrm{N}}+{\psi}_{RSA-SN}$ compared with ${\psi}_{MSA-SN}$), the focus was directed toward the significance of 3D dependencies within the domain of 3D diagnostic data.

**Table 2:** Quantitative outcomes of the proposed model (MSA-SN + RFA-SN, MSA-SN) and baseline model (DN) to highlight the significance of multilevel feature fusion and RFA-SN model.

Model	MFF	TPR	TNR	ACC	F1	AP	AR
${\psi}_{\mathrm{D}\mathrm{N}}$	û	97.17 (0.88)	88.01 (11.56)	93.46 (4.24)	93.33 (4.14)	94.12 (2.8)	92.59 (5.4)
${\psi}_{MSA-SN}$	ü	*97.66* *(0.32)*	*91.7* *(7.3)*	*95.25* *(3.01)*	*95.1* *(3.06)*	*95.53* *(2.41)*	*94.68* *(3.69)*
${\psi}_{\mathrm{M}\mathrm{S}\mathrm{A}-\mathrm{S}\mathrm{N}}+{\psi}_{RFA-SN}$ (${\psi}_{\mathrm{M}\mathrm{S}\mathrm{A}-\mathrm{S}\mathrm{N}}$)	ü	**99.78** **(0.26)**	**96.71** **(4.59)**	**98.54** **(2.0)**	**98.5** **(2.05)**	**98.77** **(1.68)**	**98.25** **(2.42)**

old: best results, Italic: second best, MFF: Multilevel Feature Fusion.

Quantitative results (Table 2) demonstrate a significant performance gain of the proposed model due to the addition of multilevel feature fusion and the RFA-SN model compared to the DN model (baseline model without multilevel fusion), with average improvements of 5.44% in ACC, 5.54% in F1, 4.94% in AP, and 6.11% in AR. Particularly noteworthy was the increase in TNR from 88.01% to 96.71%, reflecting an average TNR gain of 9.89%. This performance boost underscores the effectiveness of multilevel feature fusion, which enhances the model ability to capture and integrate features across different spatial scales. This improvement validates the rationale behind incorporating multilevel features, highlighting their crucial role in achieving superior model performance. T-test analysis further supports the statistical significance of the proposed MSA-SN model, exhibiting an average p-value of 0.0036 (p < 0.01). Moreover, the MSA-SN + RFA-SN, identified as the top-performing proposed model, demonstrated a p-value of 0.0006 (p < 0.01) when compared to the DN baseline. These considerably reduced p-values (p < 0.01) explicitly suggest that both networks exhibited significant superiority over the baseline model, achieving a confidence level of 99%.

### Ablation studies

**1) Advantage of proposed backbone with Multilevel Feature Fusion and Sequential Training:** An extensive ablation study was conducted to estimate the success of the proposed spatial backbone with multilevel feature fusion (MFF). As shown in Table 3, the performance of the proposed MSA-SN backbone improves progressively as hierarchical feature levels are progressively aggregated ($f_{4}$ → $f_{3} , f_{4}$ → $f_{2} ,f_{3} ,f_{4}$ → $f_{1} ,f_{2} ,f_{3} ,f_{4}$), representing the effectiveness of multiscale spatial representation learning for 3D imaging data. Compared with using only the deepest feature level ($f_{4}$), the complete multilevel fusion significantly increases ACC, F1, AP, and AR by 2.27%, 2.26%, 1.91%, and 2.61%, respectively. Moreover, the TNR increases substantially from 92.29% to 96.71%, yielding an overall gain of 4.42%, which confirms the importance of incorporating corresponding spatial information from multiple encoder levels. Statistical validation using a t-test further supports the consistency of these improvements, giving a significant p-value (0.0022 < 0.01) at the 99% confidence level. These results clearly demonstrate that the proposed MSA-SN backbone effectively leverages both low-level spatial details and high-level semantic representations through hierarchical feature aggregation. In contrast, the baseline DN backbone primarily relies on high-level abstract features alone, which limits its capability to capture rich multiscale contextual information.

**Table 3:** Quantitative performance comparison of our proposed backbone model (MSA-SN) versus baseline model (DN) to highlight the significance of multilevel feature fusion and RFA-SN model.

MFF	Level	TPR	TNR	ACC	F1	AP	AR
û	${{\boldsymbol{f}}}_{4}$	98.99	92.29	96.27	96.24	96.86	95.64
ü	${{\boldsymbol{f}}}_{3},{{\boldsymbol{f}}}_{4}$	98.92	94.79	97.11	97.04	97.23	96.86
ü	${{\boldsymbol{f}}}_{2}.{{\boldsymbol{f}}}_{3},{{\boldsymbol{f}}}_{4}$	*99.13*	*95.65*	*97.61*	*97.54*	*97.7*	*97.39*
ü	${{\boldsymbol{f}}}_{1}.{{\boldsymbol{f}}}_{2}.{{\boldsymbol{f}}}_{3},{{\boldsymbol{f}}}_{4}$	**99.78**	**96.71**	**98.54**	**98.5**	**98.77**	**98.25**
${\psi}_{\mathrm{M}\mathrm{S}\mathrm{A}-\mathrm{S}\mathrm{N}}+{\psi}_{RFA-SN}$(*end-to-end*)		99.15	94.93	97.29	97.09	97.14	97.04
${\psi}_{\mathrm{M}\mathrm{S}\mathrm{A}-\mathrm{S}\mathrm{N}}+{\psi}_{RFA-SN}$(*Sequential*)		**99.78**	**96.71**	**98.54**	**98.5**	**98.77**	**98.25**

Results also show improved performance based on the sequential two-step training strategy with partial freezing and fine-tuning compared to end-to-end training

bold: best results, Italic: second best, MFF: Multilevel Feature Fusion.

Furthermore, Table 3 presents an experimental comparison between end-to-end optimization and the sequential two-step training strategy for integrating the MSA-SN and RFA-SN models. In the sequential configuration, the deep dense blocks corresponding to.

${\boldsymbol{f}}_{4}$ features of level 4 of the MSA-SN backbone (approximately 80% of the backbone weights) was first trained to capture high-level spatial representations and then frozen, while the remaining multilevel transformer encoder blocks (Levels 1–3) were fine-tuned in next stage to learn low-level features ${\boldsymbol{f}}_{1} .{\boldsymbol{f}}_{2} .{\boldsymbol{f}}_{3}$. Using the same architecture and hyperparameter settings, the sequential strategy consistently improved performance compared with end-to-end training, achieving the best overall results (TPR: 99.78%, TNR: 96.71%, ACC: 98.54%, F1: 98.50%, AP: 98.77%, and AR: 98.25%). These results demonstrate that preserving stable high-level spatial representations while progressively adapting lower-level multiscale transformer features improves convergence behavior and enhances generalization compared with direct end-to-end optimization.

**2) Advantage of self-attention:** The advantage of using self-attention in the proposed model has been evaluated, and the results are presented in Table 4. The proposed RFA-SN model capitalizes on the effectiveness of self-attention mechanisms integrated within an RNN framework to efficiently process volumetric data. Table 4 shows the model performance with and without the inclusion of the self-attention layer in the RFA-SN model. The incorporation of the self-attention mechanism notably enhances the TNR, improving it from 94.68% to 96.71%, demonstrating an average improvement of 2.14%. An empirical analysis using the average t-test underscored the statistical robustness of these findings, indicated by an average p-value (0.0135 < 0.05). This signifies statistical significance at the 95% confidence level, affirming the substantial performance enhancement achieved through the integration of self-attention within the RFA-SN model. The self-attention mechanism plays a significant role in capturing long-range dependencies across a broader context, thereby enhancing the model efficacy in modeling sequential data. Furthermore, it serves as a significant feature extractor by assigning weightage to elements based on their contribution. This facilitates the automatic extraction of meaningful features, thereby enhancing the overall performance of the proposed model.

**Table 4:** Performance comparison with and without self-attention in RFA-SN model (bold: best results, SA: self-attention).

Model	TPR	TNR	ACC	F1	AP	AR
${\psi}_{\mathrm{M}\mathrm{S}\mathrm{A}-\mathrm{S}\mathrm{N}}+{\psi}_{RFA-SN}$(without SA)	99.82 (0.27)	94.68 (8.02)	97.73 (3.21)	97.72 (3.21)	98.21 (2.41)	97.25 (3.98)
${\psi}_{\mathrm{M}\mathrm{S}\mathrm{A}-\mathrm{S}\mathrm{N}}+{\psi}_{RFA-SN}$(with SA)	**99.78** **(0.26)**	**96.71** **(4.59)**	**98.54** **(2.0)**	**98.5** **(2.05)**	**98.77** **(1.68)**	**98.25** **(2.42)**

### Performance comparison with vanilla transformer

The proposed MSA-SN is compared to vanilla ViT models^36^ for the analysis of 3D imaging data using the same RFA-SN framework. Results are presented in Table 5, highlighting the remarkable performance improvements achieved by the proposed spatial backbone model compared to the vision transformer. To ensure fair comparison with modern attention-based architectures, standard ViT-tiny and ViT-small backbones were implemented under the same preprocessing pipeline, identical training/testing splits, and identical classification framework (RFA-SN) as the proposed model. Only, the CT slices were resized according to the standard input requirement of the pretrained ViT models (384 × 384), and both ViT-tiny and ViT-small employed 16 × 16 patch-based tokenization. All remaining training and optimization settings were preserved to maintain consistency and fairness across experiments. Our MSA-SN model demonstrates clear superiority over ViT-tiny, exhibiting substantial improvements of 2.45%, 2.53%, 2.54%, and 2.53% in ACC, F1, AP, and AR, respectively. It notably enhances both TPR and TNR by an average of 2.16% and 2.88%, respectively. Even against ViT-small, the MSA-SN model outperforms, showing significant improvements of 1.24%, 1.24%, 1.02%, and 1.48% in ACC, F1, AP, and AR, respectively. Furthermore, it demonstrates a remarkable improvement in TNR, increasing it from 94.14% to 96.71%, indicating an average gain of 2.73% in TNR. In t-test analysis, the MSA-SN model obtains an average p-value of 0.00001 < 0.01 and 0.0042 < 0.01, when compared to ViT-tiny and ViT-small, respectively. These significant p-values indicate the superiority of our model over both ViT models with a 99% confidence level. This design ensures that performance improvements occur from the multilevel attention-based feature aggregation and recurrent volumetric alignment strategy rather than differences in preprocessing pipelines or training configurations.

**Table 5:** Quantitative performance comparison of our proposed backbone model (MSA-SN) versus visual transformer (ViT) models in the context of 3D imaging data analysis using the same RFA-SN framework (bold: best results, underlines: second best).

Model	TPR	TNR	ACC	F1	AP	AR
$${\psi}_{{ViT}^{t}}$$$$+{\psi}_{RFA-SN}$$	97.67 (2.9)	94 (7.22)	96.18 (3.96)	96.07 (4.13)	96.32 (3.91)	95.83 (4.41)
$${\psi}_{{ViT}^{s}}$$$$+{\psi}_{RFA-SN}$$	*99.51* *(0.71)*	*94.14* *(6.29)*	*97.33* *(2.41)*	*97.29* *(2.42)*	*97.77* *(1.82)*	*96.82* *(3.02)*
${\psi}_{\mathrm{M}\mathrm{S}\mathrm{A}-\mathrm{S}\mathrm{N}}+{\psi}_{RFA-SN}$ (${\psi}_{\mathrm{M}\mathrm{S}\mathrm{A}-\mathrm{S}\mathrm{N}}$)	**99.78** **(0.26)**	**96.71** **(4.59)**	**98.54** **(2.0)**	**98.5** **(2.05)**	**98.77** **(1.68)**	**98.25** **(2.42)**

### Slice count analysis

**Fig. 4:**
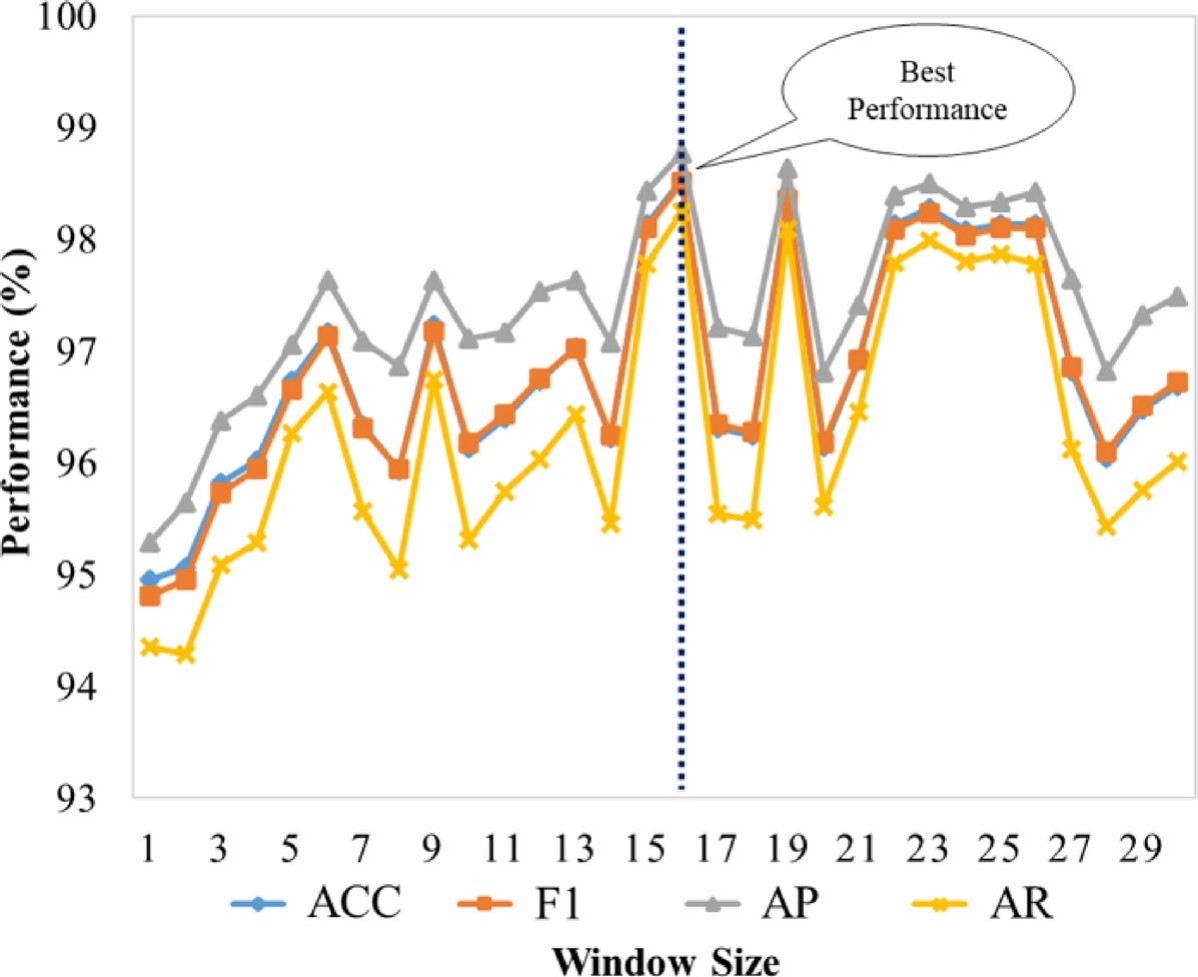
Validation results of the proposed model at various window sizes. The dotted line signifies the optimal window size 16, where the model achieves its highest performance across evaluation metrics, including ACC, F1, AP, and AR.

The proposed framework employs 2D spatial information as well as 3D structural information for diagnostic decisions. The optimal slice count was selected by analyzing the model performance at various window sizes. The window size corresponds to the number of consecutive slices considered for feature extraction. There is a trade-off between window size and model size. A small window size may result in the loss of structural information, potentially decreasing model performance. Conversely, an increase in window size leads to a minute improvement in performance but comes with a significant increase in processing time. Therefore, thirty different window sizes (depicted in Fig. 4), ranging from 1 to 30, are selected for model evaluation. The vertical dotted line in Fig. 4 indicates the peak performance, observed at a window size of 16 (w = 16), in terms of ACC, F1, AP, and AR.

The proposed framework uses an LSTM module as backbone in second-stage RFA-SN model that inherently supports variable-length slice sequences. After first stage spatial feature extraction (using MSA-SN model) from each slice, the second-stage model processes the feature sequence sequentially and aggregates contextual information through its memory mechanism. The final hidden state represents the entire volume and produces a single prediction regardless of whether the input contains one slice, 16 slices, or longer sequences.

### Comparative analysis

This section comprehensively compares the performance of the proposed models MSA-SN and MSA-SN + RFA-SN with other existing image- and sequence-based techniques, respectively. In the image-based comparison, the entire dataset was considered as 2D data, ignoring the 3D anatomical information. In the sequence-based comparison, both spatial and 3D structural information are incorporated. In the image-based analysis, Ahuja et al.^7^ achieved the second-best performance using ResNet-18. The comparison of our proposed model with their method shows substantial improvements of 2.29%, 2.34%, 2.12%, and 2.54% in ACC, F1, AP, and AR, respectively. Furthermore, it exhibited a remarkable increase in TPR and TNR, elevating it from 96.5% to 97.66% (a gain of 1.16%) and 87.77% to 91.7% (a gain of 3.93%), respectively. The t-test score indicates statistical relevance by achieving an average p-value (p < 0.01) of 0.0006 at the 99% confidence level. Likewise, Apostolopoulos et al.^37^ developed a diagnostic model based on MobileNetV2^25^. Their method is positioned as the third-best approach among other models focused on image-based analysis. The comparison shows that our proposed model gains improvements of 2.3%, 2.25%, 1.72%, and 2.74% in ACC, F1, AP, and AR, respectively. Furthermore, it exhibited a remarkable boost in TNR, elevating it from 86.61% to 91.7% (a gain of 5.09%). The t-test result demonstrates statistical significance at the 99% confidence level (p < 0.01), with an average p-value of 0.0061.

The performance of our final sequence-based model is evaluated against several sequence-based classification methods^10,13,14^ under the same experimental configuration. We compare the performance of our model with the DSS-Net method proposed by Owais et al.^13^, which is recognized as the second-best among sequence-based approaches. However, our proposed model (MSA-SN + RFA-SN) surpassed it, demonstrating average improvements of 1.96%, 1.98%, 1.7%, and 2.24% in ACC, F1, AP, and AR, respectively. It notably increased TNR from 93.01% to 96.71% (an average TNR gain of 3.7%). In the t-test analysis, our model achieved a p-value (0.0016 < 0.01), indicating that our final network significantly outperforms the baseline model at a 99% confidence level. This improvement is attributed to the enhanced capabilities of our model. Kollias et al.^10^ proposed a ResNet50 + LSTM, ranked as the third-best among the other sequence-based methods. However, our final model (MSA-SN + RFA-SN) outperformed it, with average gains of 6.4%, 6.18%, 4.37%, and 7.62% in ACC, F1, AP, and AR, respectively. Furthermore, it exhibited a remarkable boost in TPR and TNR, elevating it from 98.62% to 99.78% (a gain of 1.16%) and 82.65% to 96.71% (a gain of 14.06%), respectively. The t-test score indicates statistical significance at the 99% (p-value: 0.0063 < 0.01) confidence level.

The proposed model primarily leverages and consolidates multilevel spatial features from individual 2D input slices independently and further integrates three-dimensional structural features, thereby enabling highly accurate decisions. Consequently, our proposed model outperforms several existing methods, as indicated in Table 6.

**Table 6:** Comparative performance analysis of our proposed MSA-SN + RFA-SN (sequence-based model) and MSA-SN (image-based model) with various state-of-the-art methods (bold: best results, underlines: second best).

Study	TPR	TNR	ACC	F1	AP	AR
Hu et al.^38^	96.8	84.08	91.65	91.52	92.69	90.44
Elpeltagy et al.^8^	97.85	79.22	90.3	90.22	92.17	88.53
Wu et al.^9^	94.56	82.81	89.8	89.71	90.92	88.68
Ahuja et al.^7^	96.5	*87.77*	*92.96*	92.76	93.41	*92.14*
Aksoy et al.^39^	97.39	79.9	90.3	90.26	92.17	88.64
Apostolopoulos et al.^37^	*97.27*	86.61	92.95	*92.85*	*93.81*	91.94
Arzmi et al.^40^	*98.25*	81.74	91.56	91.51	93.13	89.99
**Proposed (**${{\boldsymbol{\psi}}}_{\mathbf{M}\mathbf{S}\mathbf{A}-\mathbf{S}\mathbf{N}}$**)**	**97.66**	**91.7**	**95.25**	**95.1**	**95.53**	**94.68**
Kollias et al.^10^	98.62	82.65	92.14	92.32	94.4	90.63
Owais et al.^13^	*99.02*	*93.01*	*96.58*	*96.53*	*97.07*	*96.01*
Gupta et al.^14^	96.99	85.26	92.23	92.27	93.59	91.13
**Proposed** $({{\boldsymbol{\psi}}}_{\mathbf{M}\mathbf{S}\mathbf{A}-\mathbf{S}\mathbf{N}+\mathbf{R}\mathbf{F}\mathbf{A}-\mathbf{S}\mathbf{N}})$	**99.78**	**96.71**	**98.54**	**98.51**	**98.77**	**98.25**

## Discussion

**Fig. 5:**
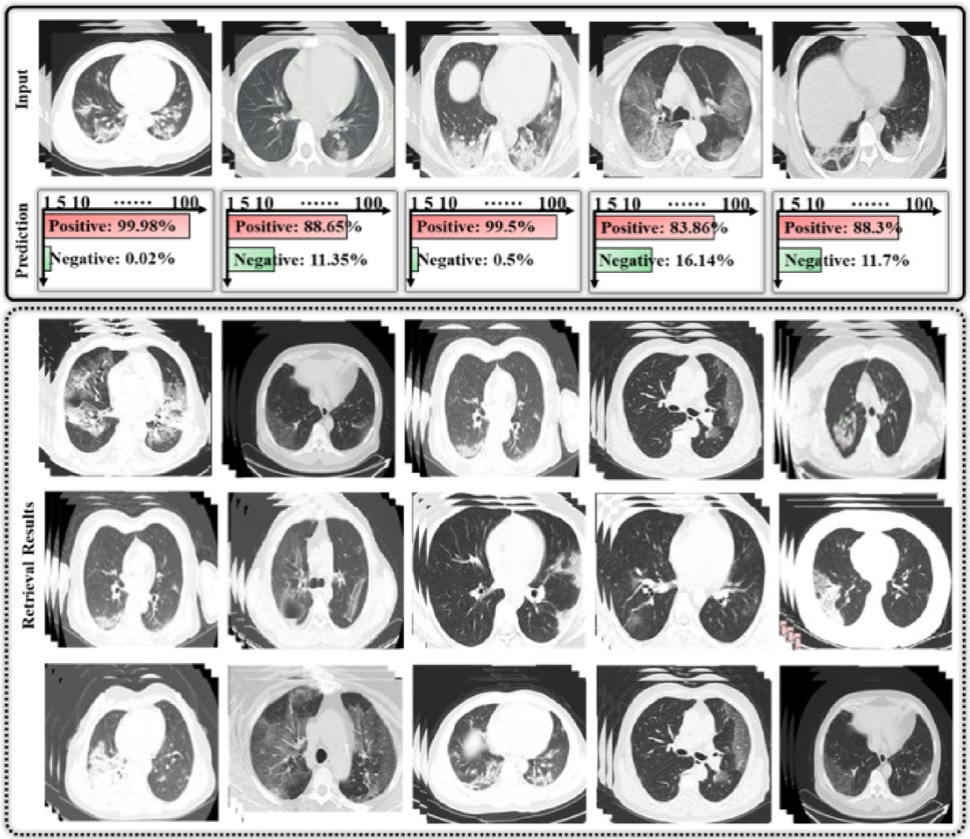
Visualization of retrieval results of the proposed model by applying cosine similarity measure. The solid line enclosed the input samples with prediction values, whereas the dotted line enclosed the retrieved results obtained by applying the cosine similarity measure.

Volumetric medical data (such as CT and MRI) contain complex three-dimensional anatomical structures. Conventional 2D CNN-based models primarily capture spatial relationships within individual slices, which may limit performance due to the lack of volumetric contextual information. In contrast, 3D CNN-based approaches manipulate full volumetric structure but require high computational resources and fixed-length inputs. To address these challenges, we propose a hybrid framework efficient of efficiently handling both 2D and 3D diagnostic imaging sequences. In the first stage, the proposed MSA-SN extracts multilevel spatial features from successive CT slices using a 2D CNN backbone. In the second stage, the RFA-SN model captures inter-slice dependencies to exploit volumetric structural information for final classification. The ablation results further confirm that transfer learning and multilevel feature fusion significantly contribute to improved classification performance (Fig. 5).

To enhance interpretability and clinical usability, the proposed framework is further extended to a volumetric medical content retrieval system using deep feature representations extracted from the trained network. In the retrieval setup, patient-independent CT slices from the validation dataset were used as retrieval database (325 infected and 222 normal images), while the testing dataset (651 infected and 443 normal images) used as the query set, ensuring no overlap with the training data and avoiding data leakage. During inference, each query scan was matched against the retrieval pool using a one-to-many similarity matching strategy based on feature-level similarity between volumetric representations. The acceptability of retrieved matches was evaluated according to class-label consistency between the query and retrieved cases, which is why classification-style performance metrics (TPR, TNR, ACC, F1, AP, AR) are reported in Table 7 instead of ranking-based retrieval metrics. Among the evaluated similarity measures, cosine similarity exhibited the best retrieval consistency compared with Euclidean and Chebyshev distances due to its effectiveness in capturing semantic relationships between deep feature embeddings.

**Table 7:** Quantitative retrieval results of the proposed model by applying three different similarity matching algorithms (bold: best results, underlines: second best).

Model	TPR	TNR	ACC	F1	AP	AR
Chebyshev	98.53 (2.52)	99.91 (0.2)	99.09 (1.48)	99.09 (1.46)	98.96 (1.69)	99.22 (1.23)
Euclidean	*98.71* *(2.26)*	*99.91* *(0.2)*	*99.20* *(1.32)*	*99.19* *(1.31)*	*99.08* *(1.52)*	*99.31* *(1.1)*
**Cosine**	**99.51** **(0.67)**	**100** **(0.0)**	**99.71** **(0.4)**	**99.70** **(0.41)**	**99.65** **(0.49)**	**99.75** **(0.34)**

**Fig. 6:**
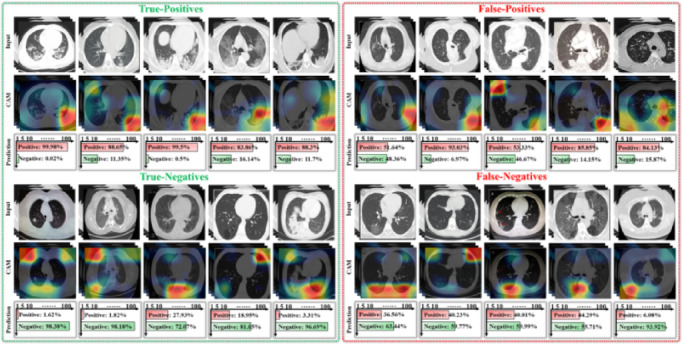
Visualization of accurate and inaccurate sample classification with the corresponding top five predicted probabilities. The green dotted line encompasses correctly classified positive and negative results, while the red dotted line encloses incorrectly detected cases.

Additionally, class activation map (CAM) visualizations (Fig. 6) illustrate the regions contributing most strongly to model predictions, highlighting the discriminatory capability of the proposed framework. These visual explanations support clinical interpretability by allowing experts to verify whether activated regions correspond to relevant pathological structures. It is important to note that the presented CAM visualizations are generated from the 2D MSA-SN backbone and therefore highlight slice-level discriminative spatial regions expended during feature extraction rather than directly describing the final volume-level prediction, which is obtained after sequential feature aggregation through the LSTM module. Since the recurrent attention mechanism operates at the feature-sequence level across slices, explicit slice-wise attention visualization in image space is not directly available. To further support qualitative interpretability, representative true-positive, true-negative, false-positive, and false-negative cases are illustrated in Fig. 6, demonstrating the model behavior across different prediction scenarios. The few remaining failure cases are mainly associated with small pathological regions and potential label noise, which remain challenging for slice-sequence modeling frameworks. Additionally, the dataset labels used in this study were expert-annotated at class-level. The objective of this work is to demonstrate how effectively the proposed AI framework can approximate expert-level decision-making, and the achieved performance of approximately 98% accuracy further supports the reliability of the qualitative activation patterns observed in the CAM visualizations.

Despite the promising results, the proposed framework has some limitations. Performance may be affected when training samples are limited for certain classes, and variations in imaging protocols across datasets may introduce intra-class variability. Future work will focus on further improving generalization through larger and more diverse annotated datasets. In addition, since volume-level labels are assigned to individual slices during training, some slice-level label noise may exist because not all slices essentially contain visible pathological regions; however, this effect is partially alleviated through appropriate data selection and LSTM-based sequential feature aggregation, and can be further addressed in future work using attention-based or weakly supervised slice-selection strategies (Fig. 6).

## Conclusion

In this work, we addressed the critical challenge of analyzing variable-length 3D medical imaging data by proposing a novel deep learning framework that eliminates the dependency on manual slice selection and expert intervention. Our approach effectively integrates 2D spatial and 3D structural features from full-length CT scans, enabling precise and robust diagnostic decisions. Extensive evaluations on publicly accessible datasets demonstrated the significant performance gains of the proposed model over existing state-of-the-art methods. This framework improves diagnostic accuracy and handles variable-length inputs efficiently, paving the way for broader clinical applicability. Future efforts should focus on integrating this method into clinical workflows to assess its practical utility and reliability in real-world scenarios. Expanding the model to incorporate multimodal data, such as 2D X-rays and patient histories, can further enhance diagnostic capabilities. Additionally, optimizing for real-time processing and advancing model interpretability will be key to fostering adoption and trust among healthcare professionals.

## Data Availability

This study uses three publicly available CT datasets: BIMCV COVID-19^43^, COVID-CT^44^, and The Cancer Imaging Archive (TCIA)^45^. The proposed framework will be publicly released on our GitHub repository to support further research.

## Abbreviations

3D: Three-dimensional
2D: Two-dimensional
CNN: Convolutional neural network
CAD: Computer-aided diagnosis
CT: Computed tomography
MRI: Magnetic resonance imaging
OCT: Optical coherence tomography
LSTM: Long short-term memory
ViT: Vision transformer
MSR-AN: Multilevel spatio-recurrent attention network
MSA-SN: Multilevel spatio-attention sub-network
RFA-SN: Recurrent feature alignment sub-network
AP: Average precision
AR: Average recall
ACC: Accuracy
F1: F1-Score
TPR: True positive rate
TNR: True negative rate
FC: Fully connected
SA: Self-attention

## References

[1] Chen, B. et al. Trends and hotspots in research on medical images with deep learning: a bibliometric analysis from 2013 to 2023. *Front. Artif. Intell.* **6**, 1289669 (2023). 38028662 10.3389/frai.2023.1289669 PMC10665961

[2] Jiang, H., Tang, S., Liu, W. & Zhang, Y. Deep learning for COVID-19 chest CT (computed tomography) image analysis: A lesson from lung cancer. *Comput. Struct. Biotechnol. J.* **19**, 1391–1399 (2021). 33680351 10.1016/j.csbj.2021.02.016 PMC7923948

[3] Owais, M., Hassan, T., Gour, N., Ganapathi, I. I. & Werghi, N. Strengthening Deep Learning Model for Robust Screening of Volumetric Chest Radiographic Scans. In *2023 IEEE International Conference on Image Processing (ICIP)*, pp. 1545-1549 (IEEE, 2023).

[4] Kudo, Y. et al. AI-driven characterization of solid pulmonary nodules on CT imaging for enhanced malignancy prediction in small-sized lung adenocarcinoma. *Clin. Lung Cancer* **25**(5), 431–439 (2024). 10.1016/j.cllc.2024.04.015 38760224

[5] Lancaster, H. L., Heuvelmans, M. A. & Oudkerk, M. Low-dose computed tomography lung cancer screening: Clinical evidence and implementation research. *J. Intern. Med.* **292**(1), 68–80 (2022). 10.1111/joim.13480 PMC9311401 35253286

[6] Yang, Y., Li, X., Fu, J., Han, Z. & Gao, B. 3D multi-view squeeze-and-excitation convolutional neural network for lung nodule classification. *Med. Phys.* **50**(3), 1905–1916 (2023). 36639958 10.1002/mp.16221

[7] Ahuja, S., Panigrahi, B. K., Dey, N., Rajinikanth, V. & Gandhi, T. K. Deep transfer learning-based automated detection of COVID-19 from lung CT scan slices. *Appl. Intell.* **51**(1), 571–585 (2021). 10.1007/s10489-020-01826-w PMC7440966 34764547

[8] Elpeltagy, M. & Sallam, H. Automatic prediction of COVID−19 from chest images using modified ResNet50. *Multimed. Tools Appl.* **80**(17), 26451–26463 (2021). 33967592 10.1007/s11042-021-10783-6 PMC8095476

[9] Wu, Y. et al. A vision transformer for emphysema classification using CT images. *Phys. Med. Biol.* **66**(24), 245016 (2021). 10.1088/1361-6560/ac3dc8 34826824

[10] Kollias, D., Arsenos, A. & Kollias, S. Ai-mia: Covid-19 detection and severity analysis through medical imaging. In *European Conference on Computer Vision (ECCV)*, 677–690 (Springer Nature Switzerland, Cham, 2022).

[11] Bui, N. T. et al. Sam3d: Segment anything model in volumetric medical images. In *2024 IEEE International Symposium on Biomedical Imaging (ISBI)*, pp. 1-4 (IEEE, 2024).

[12] Arsenos, A., Kollias, D. & Kollias, S. A large imaging database and novel deep neural architecture for covid-19 diagnosis. In *2022 IEEE 14th Image, Video, and Multidimensional Signal Processing Workshop (IVMSP)*, pp. 1-5 (IEEE, 2022).

[13] Owais, M. et al. Deep 3D volumetric model genesis for efficient screening of lung infection using chest CT scans. *Mathematics* **10**(21), 4160 (2022).

[14] Gupta, S., Garg, A., Bishnoi, V. & Goel, N. Pulmonary nodules binary classification using cnn and lstm. In *2023 10th International Conference on Signal Processing and Integrated Networks (SPIN)*, pp. 174-179 (IEEE, 2023).

[15] Zhang, Y. et al. PB-LNet: a model for predicting pathological subtypes of pulmonary nodules on CT images. *BMC Cancer* **23**(1), 936 (2023). 37789252 10.1186/s12885-023-11364-6 PMC10548640

[16] Vaswani, A. et al. Attention is all you need. In *Advances in Neural Information Processing Systems*, 30 (2017).

[17] Kaur, T. & Gandhi, T. K. Automated brain image classification based on VGG-16 and transfer learning. In *2019 International Conference on Information Technology (ICIT)*, pp. 94-98 (IEEE, 2019).

[18] Simonyan, K. & Zisserman, A. Very deep convolutional networks for large-scale image recognition. *arXiv preprint* arXiv:1409.1556 (2014).

[19] Ashraf, R. et al. Deep convolution neural network for big data medical image classification. *IEEE Access* **8**, 105659–105670 (2020).

[20] Szegedy, C. et al. Going deeper with convolutions. In *Proceedings of the IEEE Conference on Computer Vision and Pattern Recognition (CVPR)*, pp. 1–9 (2015).

[21] Akpinar, K. N., Genc, S. & Karagol, S. Chest x-ray abnormality detection based on squeezenet. In *2020 International Conference on Electrical, Communication, and Computer Engineering (ICECCE)*, pp. 1–5 (IEEE, 2020).

[22] Zhang, X., Zhou, X., Lin, M. & Sun, J. Shufflenet: An extremely efficient convolutional neural network for mobile devices. In *Proceedings of the IEEE Conference on Computer Vision and Pattern Recognition (CVPR)*, pp. 6848–6856 (2018).

[23] Aloyayri, A. & Krzyżak, A. Breast cancer classification from histopathological images using transfer learning and deep neural networks. In *International Conference on Artificial Intelligence and Soft Computing*, pp. 491–502 (Springer International Publishing, Cham, 2020).

[24] Souid, A., Sakli, N. & Sakli, H. Classification and predictions of lung diseases from chest x-rays using mobilenet v2. *Appl. Sci.* **11**(6), 2751 (2021).

[25] Sandler, M., Howard, A., Zhu, M., Zhmoginov, A. & Chen, L. C. Mobilenetv2: Inverted residuals and linear bottlenecks. In *Proceedings of the IEEE Conference on Computer Vision and Pattern Recognition (CVPR)*, pp. 4510-4520 (2018).

[26] Jasil, S. G. & Ulagamuthalvi, V. Skin lesion classification using pre-trained DenseNet201 deep neural network. In *2021 3rd International Conference on Signal Processing and Communication (ICPSC)*, pp. 393–396 (IEEE, 2021).

[27] Çakmak, M. & Tenekecı, M. E. Melanoma detection from dermoscopy images using Nasnet Mobile with Transfer Learning. In *2021 29th Signal Processing and Communications Applications Conference (SIU)*, pp. 1–4 (IEEE, 2021).

[28] Huang, G., Liu, Z., Van Der Maaten, L. & Weinberger, K. Q. Densely connected convolutional networks. In *Proceedings of the IEEE Conference on Computer Vision and Pattern Recognition (CVPR)*, pp. 4700–4708 (2017).

[29] Zoph, B., Vasudevan, V., Shlens, J. & Le, Q. V. Learning transferable architectures for scalable image recognition. In *Proceedings of the IEEE Conference on Computer Vision and Pattern Recognition (CVPR)* 8697–8710 (2018) .

[30] Gambhir, R., Bhardwaj, S., Kumar, A. & Agarwal, R. Severity classification of diabetic retinopathy using ShuffleNet. In *2021 International Conference on Intelligent Technologies (CONIT)*, pp. 1–5 (IEEE, 2021).

[31] Srinivasu, P. N. et al. Classification of skin disease using deep learning neural networks with MobileNet V2 and LSTM. *Sensors* **21**(8), 2852 (2021). 33919583 10.3390/s21082852 PMC8074091

[32] Ebrahimi, A., Luo, S., Chiong, R. & Alzheimer’s Disease Neuroimaging Initiative. Deep sequence modelling for Alzheimer’s disease detection using MRI. *Comput. Biol. Med.* **134**, 104537 (2021). 34118752 10.1016/j.compbiomed.2021.104537

[33] He, K., Zhang, X., Ren, S. & Sun, J. Deep residual learning for image recognition. In *Proceedings of the IEEE Conference on Computer Vision and Pattern Recognition (CVPR)*, pp. 770–778 (2016).

[34] Shahzadi, I., Tang, T. B., Meriadeau, F. & Quyyum, A. CNN-LSTM: Cascaded framework for brain tumour classification. *In 2018 IEEE-EMBS Conference on Biomedical Engineering and Sciences (IECBES) *, pp. 633–637 (IEEE, 2018).

[35] Abdar, M. et al. Hercules: Deep hierarchical attentive multilevel fusion model with uncertainty quantification for medical image classification. *IEEE Trans. Ind. Inform.*, **19**(1), 274–285 (2022).

[36] Dosovitskiy, A. et al. An image is worth 16x16 words: Transformers for image recognition at scale. *arXiv preprint* arXiv:2010.11929 (2020).

[37] Apostolopoulos, I. D., Apostolopoulos, D. J. & Papathanasiou, N. D. Deep learning methods to reveal important X-ray features in COVID-19 detection: Investigation of explainability and feature reproducibility. *Reports* **5**(2), 20 (2022).

[38] Hu, R. et al. Automated diagnosis of covid-19 using deep learning and data augmentation on chest ct. *Medrxiv*, pp. 2020-04 (2020).

[39] Aksoy, B. & Salman, O. K. M. Prediction of Covid-19 disease with Resnet-101 deep learning architecture using Computerized Tomography images. *Türk Doğa ve Fen Dergisi* **11**(2), 36–42 (2022).

[40] Arzmi, M. H. et al. The Classification of Lung Cancer: A DenseNet Feature-Based Transfer Learning Evaluation. In *Deep Learning in Cancer Diagnostics: A Feature-based Transfer Learning Evaluation *, pp. 21-26 (Springer Nature Singapore, Singapore, 2023).

[41] Cao, W., Hussain, N. & Yan, Z. RDNet: Region specific iterative deformation with multi-scale attention for medical image registration. *Neurocomputing* **658**, 131455 (2025).

[42] Hussain, N., Yan, Z., Cao, W. and Anwar, M. Hierarchical refinement with adaptive deformation cascaded for multi-scale medical image registration. *Magn. Reson. Imaging* **122**, 110449 (2025). 40541621 10.1016/j.mri.2025.110449

[43] Vayá, M. D. L. I. et al. BIMCV COVID-19+: a large annotated dataset of RX and CT images from COVID-19 patients. *arXiv preprint* arXiv:2006.01174 (2020).

[44] Yang, X. et al. Covid-ct-dataset: a ct scan dataset about covid-19. *arXiv preprint* arXiv:2003.13865 (2020).

[45] Clark, K. et al. The Cancer Imaging Archive (TCIA): maintaining and operating a public information repository. *J. Digit. Imaging.* **26**(6), 1045–1057 (2013). 23884657 10.1007/s10278-013-9622-7 PMC3824915

[46] Sun, G. et al. DA-TransUNet: Integrating spatial and channel dual attention with transformer U-net for medical image segmentation. *Front. Bioeng. Biotechnol.* **12**, 1398237 (2024). 38827037 10.3389/fbioe.2024.1398237 PMC11141164

[47] Pan, Y. et al. A mutual inclusion mechanism for precise boundary segmentation in medical images. *Front. Bioeng. Biotechnol.* **12**, 1504249 (2024). 39777107 10.3389/fbioe.2024.1504249 PMC11704489

[48] Sun, G. et al. Fkd-med: privacy-aware, communication-optimized medical image segmentation via federated learning and model lightweighting through knowledge distillation. *Ieee Access* **12**, 33687–33704 (2024).

